# Monocarboxylate transporter 1 and monocarboxylate transporter 4 in cancer-endothelial co-culturing microenvironments promote proliferation, migration, and invasion of renal cancer cells

**DOI:** 10.1186/s12935-019-0889-8

**Published:** 2019-06-28

**Authors:** Chen Guo, Tao Huang, Qing-Hai Wang, Hong Li, Aashish Khanal, En-Hao Kang, Wei Zhang, Hai-Tao Niu, Zhen Dong, Yan-Wei Cao

**Affiliations:** 1 Department of Urology, The Affiliated Hospital of Qingdao University, No. 59 Haier Road, Qingdao, 266071 Shandong China; 2 Department of Pathology, The Affiliated Hospital of Qingdao University, Qingdao, Shandong China; 3 Department of Pathology, 401 Hospital of People’s Liberation Army, Qingdao, Shandong China

**Keywords:** Monocarboxylate transporter, Glycolytic metabolism, Renal cell carcinoma, Cancer-endothelial microenvironment, Cancer invasion, Cancer metastasis

## Abstract

**Background:**

The Warburg effect demonstrates the importance of glycolysis in the development of primary and metastatic cancers. We aimed to explore the role of monocarboxylate transporter 1 (MCT1) and MCT4, two essential transporters of lactate, in renal cancer progression during cancer-endothelial cell co-culturing.

**Methods:**

Renal cancer cells (786-O) and human vascular endothelial cells (HUVECs) were single-cultured or co-cultured in transwell membranes in the presence or absence of a MCT-1/MCT-4 specific blocker, 7ACC1. Cell proliferation was evaluated with the CCK-8 kit, while cell migration, after a scratch and invasion in transwell chambers, was evaluated under a microscope. Real-time qPCR and western blot were employed to determine the mRNA and protein levels of MCT1 and MCT4, respectively. The concentration of lactic acid in the culture medium was quantified with an l-Lactic Acid Assay Kit.

**Results:**

786-O cells and HUVECs in the co-culturing mode exhibited significantly enhanced proliferation and migration ability, compared with the cells in the single-culturing mode. The expression of MCT1 and MCT4 was increased in both 786-O cells and HUVECs in the co-culturing mode. Co-culturing promoted the invasive ability of 786-O cells, and markedly increased extracellular lactate. Treatments with 7ACC1 attenuated cell proliferation, migration, and invasion, and down-regulated the levels of MCT1/MCT4 expression and extracellular lactate.

**Conclusions:**

The Warburg effect accompanied with high MCT1/MCT4 expression in the cancer-endothelial microenvironments contributed significantly to renal cancer progression, which sheds new light on targeting MCT1/MCT4 and glycolytic metabolism in order to effectively treat patients with renal cancers.

## Background

Renal cell carcinoma (RCC) is the most common malignancy that originates in kidney, and it contributes to nearly 2.4% of whole cancer burden and 1.7% of all cancer-related global deaths [1]. RCC is derived from the proximal tubular epithelial cells of the renal cortex and accounts for approximately 85% to 90% of kidney cancers [2]. The pathology of RCC is featured with a high level of vascularization, and any variation in the vascularity can contribute to alterations in the progression of this disease [3]. As the main cell type of tumor vessels, vascular endothelial cells (ECs) play critical roles in angiogenesis and are essential for the growth, proliferation, and migration of cancer cells [4]. Therefore, disrupting the interaction between ECs and RCC in the tumor microenvironment is considered as an effective strategy in treating patients with RCC [5, 6].

In solid tumor cells, the interplay between tumor cells and stromal cells promotes the critical processes of angiogenesis, and tumor invasion [7, 8]. In particular, the interaction between cancer cells and endothelial cells is indispensable for cancer cell intravasation and migration crossing the endothelial barriers [9]. Furthermore, most sporadic RCCs in humans carry inactivating mutations in the VHL tumor suppressor gene, which leads to constitutive stabilization of the hypoxia inducible transcription factors HIF-1α and HIF-2α [10]. These transcription factors activate a cellular response which induces metabolic reprogramming towards aerobic glycolysis and promotes angiogenesis [11]. Therefore, identifying the metabolic enzymes that are particularly critical for RCC proliferation and invasion will help develop novel therapeutic targets and enhance the efficacy of common therapeutic agents.

The Warburg effect describes a new metabolic way in which most cancer cells function utilizing energy generated from efficient glycolysis followed by the fermentation of lactic acid and acidification [12, 13]. Enhanced glycolysis is tightly associated with tumorigenesis and metastasis [13]. Monocarboxylate transporters (MCTs) are a family of proteins that regulate glycolysis, and they are responsible for transporting lactate inside and outside of cells. MCT1 and MCT4 are two major regulators of lactate transportation under physiological and pathological conditions [14]. Accumulating studies in tumors of various types have demonstrated the significant correlations between elevated MCT1 and MCT4 expression in tumors and poor prognosis of patients [3, 15–20]. For example, we have demonstrated that the overexpression of MCT1 and MCT4 are associated with poor patient prognosis with clear cell renal cell carcinoma (ccRCC) [3]. However, the role of MCT1 and MCT4 in the proliferation and invasion of RCC has not been elucidated so far.

In this study, we established an in vitro co-culturing model of human renal clear cancer cell line 786-O and human umbilical vein endothelial cell line HUVEC and evaluated the effect of MCTs on the proliferation, migration, and invasion of 786-O cells using the specific MCTs blocker 7ACC1. In addition, the expression levels of MCT1 and MCT4 in both 786-O cells and HUVECs, as well as the extracellular levels of lactate, were compared when cells were under single-culturing and co-culturing conditions and in the presence or absence of 7ACC1 treatments. Our study suggests that MCT1 and MCT4 are key moderators of glycolysis and contribute significantly to the proliferation and invasive ability of renal tumor cells.

## Methods

### Cell culture and transwell^®^ coculture

Human renal clear cancer cell line 786-O and human umbilical vein endothelial cell line HUVEC were originally purchased from Yipu Biotechnology Company, Wuhan, China. The cells were stored in the Central Laboratory of the Affiliated Hospital of Qingdao University, Qingdao, Shandong province, China. The 786-O cells and HUVECs were routinely maintained in RPMI (Roswell Park Memorial Institute) 1640 medium supplemented with 10% fetal bovine serum (FBS; Invitrogen, USA) and 100 units/ml of penicillin and 100 µg/ml of streptomycin (Invitrogen, USA) in a humidified atmosphere with 5% CO_2_ at 37 °C.

The transwell chambers (Corning, USA) were used to establish the 786-O and HUVEC co-culture model. Since the area of the upper chamber is 1/4 of the area of the lower chamber, 1 × 10^4^ cells/well were seeded in the upper chamber, while 4 × 10^4^ cells/well were seeded in the lower chamber. The upper and lower compartment was separated by the polycarbonate membrane, which allowed the free circulation of various cytokines and metabolites secreted by the cells between the lower and upper chambers. A polycarbonate membrane with a pore diameter of 8 μm was used for the cell migration and invasion test.

786-O cells and HUVECs were cultured in the transwell chambers in the following manner: for the 786-O control, 786-O cells were seeded in both the upper and lower chambers; for the HUVEC control, HUVECs were added in both chambers; for the HUVEC coculture, 786-O cells were added in the upper chamber while HUVECs were added in the lower chamber; and for the 786-O coculture, HUVECs were added in the upper chamber while 786-O cells were added in the lower chamber. As an inhibitor that selectively interferes with lactate fluxes in the lactate-rich tumor microenvironment to suppress MCT1 and MCT4, 7ACC1 (MedChemExpress, Shanghai, China) at a concentration of 10 µM was added in each culturing condition and cells were incubated for 24 h with 7ACC1.

### Cell proliferation assays

The cell proliferation was quantitated with the Cell Counting Kit-8 (CCK-8; Beyotime, Shanghai, China) following the manufacturer’s instructions. 786-O cells and HUVECs were seeded into 24-well transwell plates (Corning, USA) and incubated with or without 10 µM 7ACC1. 200 µl from the lower chamber cell suspension was transferred into 96-well plates at 24, 48, 72, and 96 h after incubation. 50 µl of the CCK-8 reagent was then added to each well and the plates were incubated at 37 °C for an additional 2 h. The optical densities (ODs) at 450 nm were determined using a microplate reader. Triplicated wells were used for each group.

### Transwell migration and invasion assays

786-O cells (5 × 10^5^ cells/well) and HUVECs (5 × 10^5^ cells/well) were seeded into the Transwell insert in the manner mentioned above and were allowed to adhere overnight. Cell monolayers were washed with phosphate-buffered saline (PBS) and a “wound” was generated by scratching with a plastic pipette tip. At 0 and 24 h after the initiation of scratching, the scratched areas were photographed by phase contrast microscopy. The migration ability was quantified by calculating distance changes relative to the control groups using the Image J software (version 18.0; National Institutes of Health, USA). Each experiment was performed for 3 times with triplicated wells in each group.

The invasion of 786-O cells was evaluated by the Boyden chamber invasion assay using 8-micron Transwell filters. 786-O cells at a density of 1 × 10^4^ cells/well were seeded into the upper chamber in serum-free medium supplemented with or without 10 µM 7ACC1, while HUVECs were cultured in the lower chamber with complete medium containing 10% FBS. At 24 h after incubation, cells that did not migrate through the pores were removed using a cotton swab. Cells that penetrated the membrane were then counted under a microscope (Olympus Corp. Tokyo, Japan). Five random fields per chamber were selected for each group. Each experiment was performed 3 times.

### Western blotting analysis

The cells were lysed with radioimmunoprecipitation assay (RIPA) buffer for 15 min at 4 °C and cell lysates were collected after centrifugation. The protein concentration of cell lysates was determined with a bicinchoninic acid (BCA) Protein Assay kit (Abcam, UK). After separation by 10% sodium dodecyl sulfate polyacrylamide gel electrophoresis (SDS-PAGE), samples were transferred on a polyvinylidene difluoride (PVDF) membrane, and blocked with 5% bovine serum albumin (BSA) in tris-buffered saline containing 0.1% Tween 20 (TBST) at room temperature for 1 h. The membranes were incubated at 4 °C overnight with mouse monoclonal anti-MCT1, anti-MCT4, and anti-GAPDH (glyceraldehyde-3-phosphate dehydrogenase) antibodies. All antibodies were purchased from Santa Cruz Biotechnology, USA. GAPDH was used to indicate the loading amount of total protein. After washing three times with TBST, the membranes were incubated with horseradish peroxidase (HRP)-conjugated secondary goat anti-mouse antibody for 1 h. The proteins of interest were visualized using enhanced chemiluminescence (ECL) reagent (Goodbio, Wuhan, China). The intensity of the bands was quantified by densitometry using the Image J software (version 18.0).

### Reverse transcription-quantitative polymerase chain reaction (RT-qPCR)

Total RNA was extracted using the Trizol reagent (Invitrogen, USA) as per the instruction of the manufacturer’s manual. The extracted RNA was quantified using a NanoDrop device (ThermoFisher Scientific) and stored at − 80 °C pending subsequent analysis. The PrimeScript RT Reagent Kit (Takara, Japan) was used for reverse transcription of total RNA into complementary DNA (cDNA). The expression level of MCT1 and MCT4 were assessed by RT-qPCR using the SYBR^®^premix ExTaqTM II PCR Kit (Takara, Japan). The primers were synthesized by Sangon, Shanghai, China, and the sequences are as follows:

- MCT1, Forward, 5′-TGGATGGAGAGGAAGCTTTCTAAT-3′.
- Reverse, 5′-CACACCAGATTTTCCAGCTTTC-3′.
- MCT4, Forward, 5′-CACGGCATCGTCACCAACT-3′.
- Reverse, 5′-ACAGCCTGGATAGCAACGTACAT-3′.
- GAPDH, Forward, 5′-CGGAGTCAACGGATTTGGTCGTAT-3′.
- Reverse, 5′-AGCCTTCTCCATGGTGGTGAAGAC-3′.

GAPDH was the housekeeping gene used for the control. The experiments were performed 3 times and triplicated wells were used in each group in one experiment. The reaction conditions for PCR were set as follows: pre-denaturation at 95 °C for 30 s, 45 cycles of denaturation at 95 °C for 5 s, and annealing at 58 °C for 34 s. The 7500 Real-Time PCR System (Thermo Fisher Scientific) was used to perform the assay. The 2^−ΔΔCt^ method was used to calculate the expression of the target gene between the experimental group and the control group.

### Lactate measurement

The metabolic behavior of the cells under the different treatment conditions was determined by analyzing extracellular lactate. The 786-O cells and HUVECs were plated in 24-well transwell plates in the manner as described above and cultured for 96 h. Lactate was quantified using the l-Lactic Acid (l-Lactate) Assay Kit (Megazyme, Ireland) following the manufacturer’s instructions. Briefly, samples were collected after centrifugation of the culture medium. The lactic acid concentration calibration reagent was added to the supernatant samples, and samples were incubated for 5 min at 37 °C. The OD values at 340 nm were obtained from a plate reader and the concentration of lactate was calculated following the formula specified in the manual.

### Statistical analysis

All Data are expressed as mean ± standard deviation, and statistics were analyzed with Graphpad Prism (version 6.01; GraphPad Software, Inc, La Jolla, CA, USA). One-way analysis of variance (ANOVA) and Student’s test were employed to analyze the differences between the groups. A *P* value less than 0.05 was considered statistically significant.

## Results

### Both 786-O Cells and HUVECs Had Significantly Higher Viability in the Co-culture Mode Compared with Single-culture Mode

**Fig. 1:**
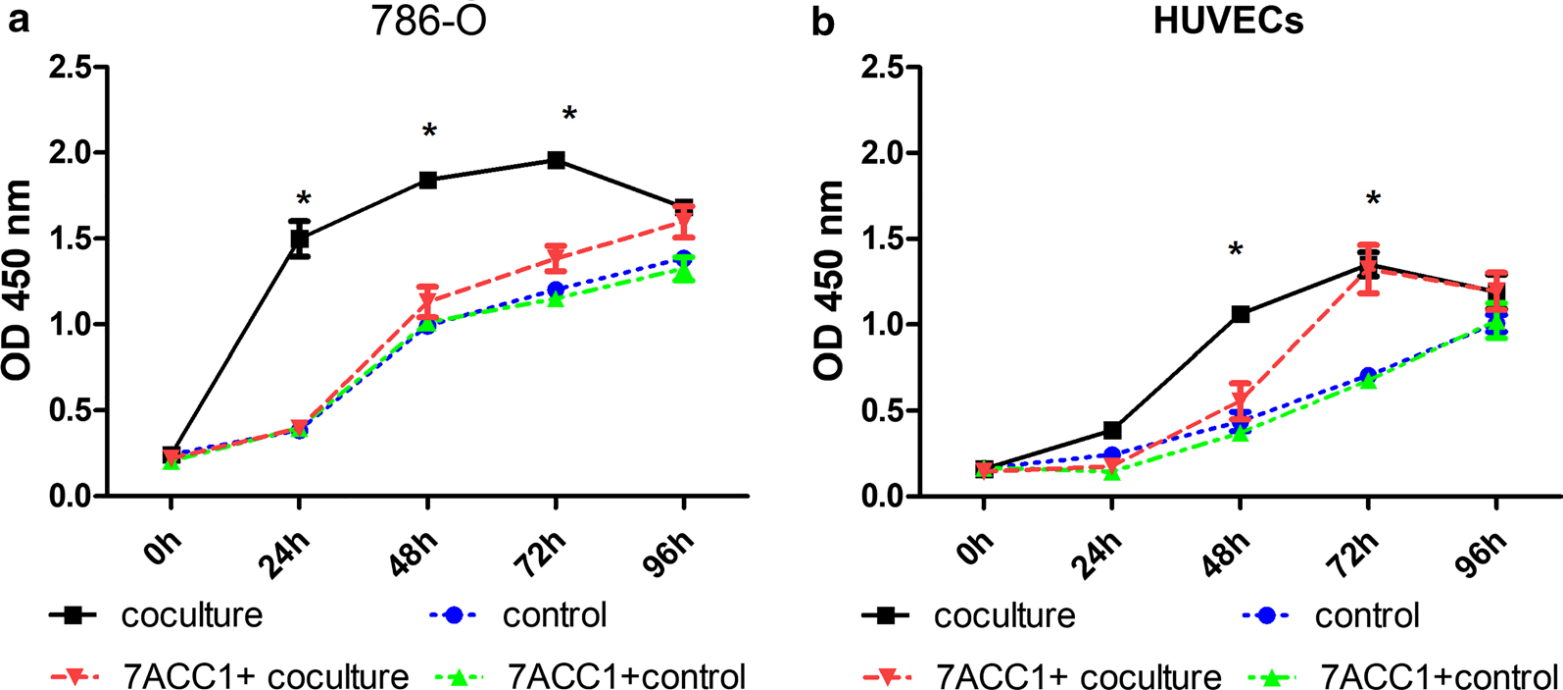
The viability of 786-O cells and HUVECs in the co-culture mode and the control single-culture mode. **a**, **b** In the transwell culturing, 1 × 10^4^ cells were seeded in the upper chamber and 4 × 10^4^ cells were seeded in the lower chamber. The viability of (**a**) 786-O cells and (**b**) HUVECs was measured by a CCK-8 assay at 0, 24, 48, 72, and 96 h after culturing. For the control, the cells were seeded in both the upper and lower chambers; for the HUVEC coculture, 786-O cells were added to the upper chamber while HUVECs were added to the lower chamber; for the 786-O coculture, HUVECs were added to the upper chamber while 786-O cells were added to the lower chamber; and, for the control + 7ACC1 or coculture + 7ACC1, 10 µM 7ACC1 was added to the culturing conditions. *P < 0.001, compared with the control

To test the in vitro role of MCT1 and MCT4 under the single-culture or co-culture conditions of 786-O cells or HUVECs, cell proliferation was determined by measuring viability via the CCK-8 assay. The single-cultured 786-O cells or HUVECs were controls. When 786-O cells and HUVECs cells were co-cultured, the viability of 786-O cells was significantly higher than that in control culturing at 24, 48, and 72 h after co-culturing (*P *< 0.001; Fig. 1a). The viability of HUVECs was also significantly higher at 48 h and 72 h in the co-culturing condition than in the control culturing condition (*P *< 0.001; Fig. 1b). The addition of MCT blocker 7ACC1 in the culture medium remarkably attenuated the differences in the viability between the control culturing and co-culturing conditions in 786-O cells at 24, 48, and 72 h and in the HUVECs at 48 h after co-culturing (*P *< 0.001; Fig. 1). However, the suppressive effect of 7ACC1 on the viability of HUVECs co-cultured for 72 h was not observed. In addition, 7ACC1 did not exert anti-proliferative effect in either 786-O cells or HUVECs in single-culturing conditions (Fig. 1). Taken together, these results suggested that co-culturing of 786-O cells and HUVECs markedly enhanced the proliferation of both cell lines, which was at least partially dependent on MCTs secreted into the culture medium.

### Co-culturing promoted the migration capacity of both 786-O cells and HUVECs and invasion ability of 786-O cells in a MCT-dependent manner

**Fig. 2:**
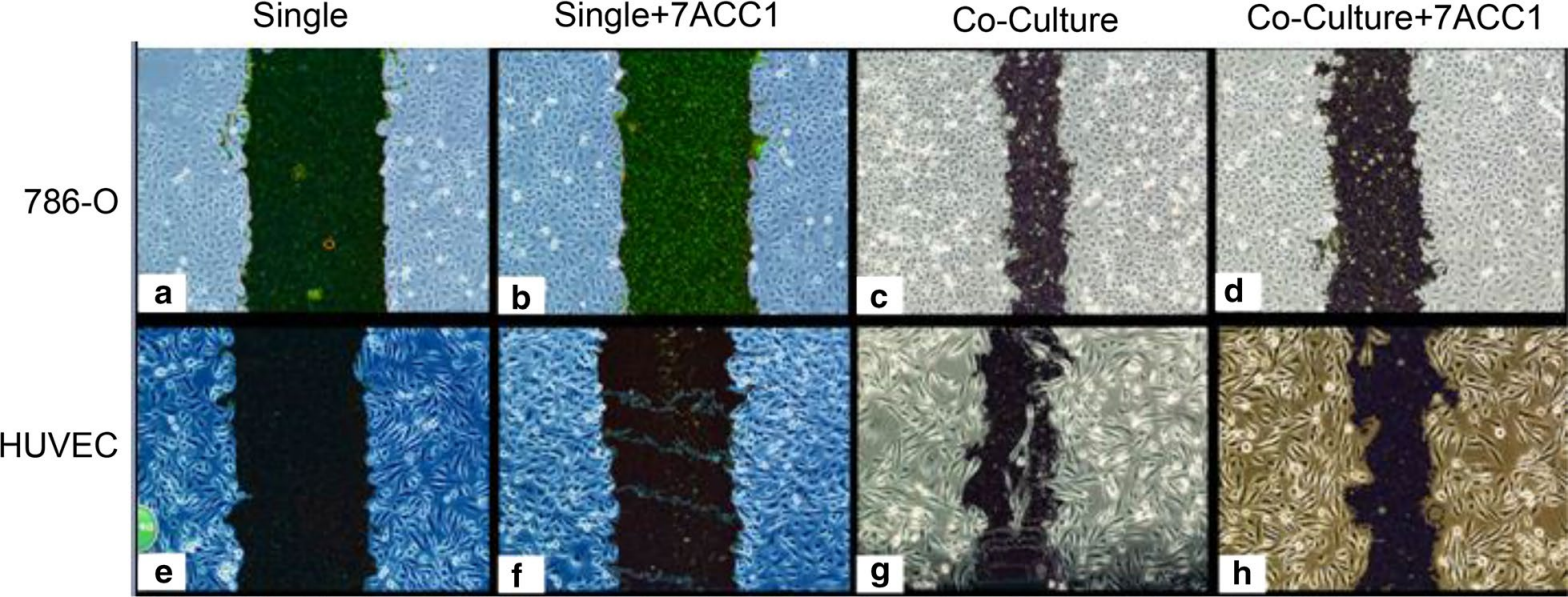
Co-culturing promoted the migration capacity of 786-O cells and HUVECs in an MCTs-dependent manner. **a**–**h** In the transwell culturing, 1 × 10^4^ cells were seeded in the upper chamber and 4 × 10^4^ cells were seeded in the lower chamber. The migration ability of 786-O cells (**a**–**d**) and HUVECs (**e**–**h**) was measured by evaluating the width of scratches at 24 h after culturing in the single-culturing mode and co-culturing mode. In the single culture, the cells were seeded in both the upper and lower chambers; in the HUVEC coculture, 786-O cells were added to the upper chamber while HUVECs were added to the lower chamber; in the 786-O coculture, HUVECs were added to the upper chamber while 786-O cells were added to the lower chamber; in Single + 7ACC1 or coculture + 7ACC1, 10 µM 7ACC1 was added to the culturing conditions

**Fig. 3:**
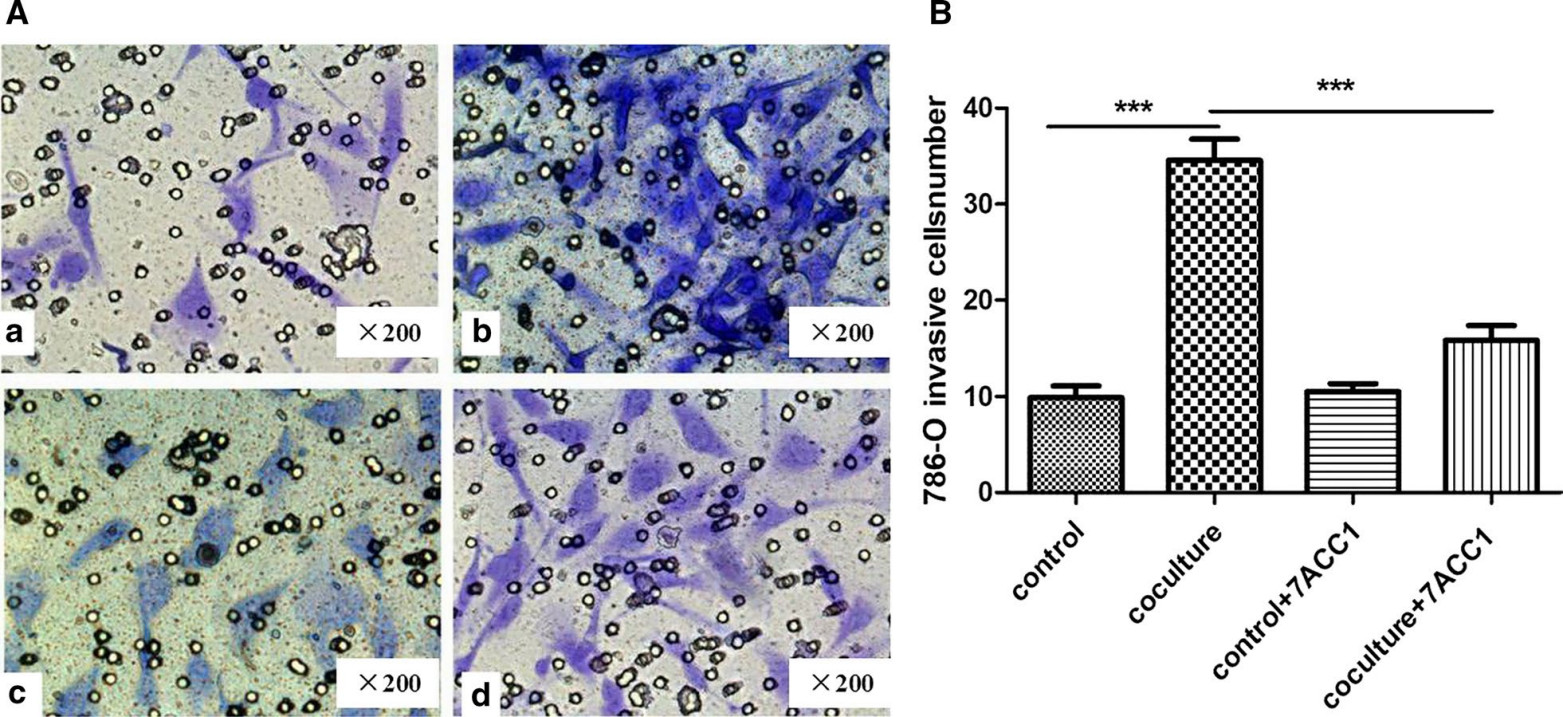
Co-culturing promoted the invasion ability of 786-O cells in an MCT-dependent manner. **A**, **B** In the transwell chamber invasion assay, 786-O cells at a density of 1 × 10^4^ cells/well were seeded in the upper chamber in serum-free medium supplemented with or without 10 µM 7ACC1, while 786-O cells or HUVECs at a density of 4 × 10^4^ cells/well were seeded in the lower chamber in complete culture medium. 24 h after culturing, the 786-O cells that penetrated the membrane were counted under a microscope. **A** Representative images show the membrane-invaded 786-O cells (magnification of ×200). **B** Summarized results on the cell number of membrane-invaded 786-O cells in each culturing condition. For the control, the 786-O cells were seeded in both the upper and lower chambers; in the co-culture, 786-O cells were added to the upper chamber while HUVECs were added to the lower chamber. ***P < 0.001, between the indicated groups

In order to evaluate if MCT1 and MCT4 can influence the migration abilities of renal cancer cells and endothelial cells, 786-O cells and HUVECs seeded in the transwell chambers in single-culturing mode or co-culturing mode were subjected to the “wound heal” test. As shown in Fig. 2, at 24 h after culturing, both 786-O cells and HUVECs showed better healing in co-culturing mode than that in single-culturing mode. Blocking MCT1 and MCT4 by supplementation of 7ACC1 in the culture medium markedly decreased migration of both 786-O cells (Fig. 2c, d) and HUVECs (Fig. 2g, h) in co-culturing mode, but it did not affect migration of cells in the single-culturing mode (Fig. 2a, b and Fig. 2e, f). To evaluate the invasion ability of renal cancer cells, the number of 786-O cells that penetrated the membrane was counted under a microscope (Fig. 3). More invasive 786-O cells were found on the surface of the lower chamber in co-culturing mode (Fig. 3b), compared to that in single–culturing condition (Fig. 3a). Remarkably, blocking of MCT1 and MCT4 by 7ACC1 treatment significantly decreased the invasive 786-O cells in the co-culture model (*P *< 0.001; Fig. 3d) but not 786-O cells in the single-culturing mode (Fig. 3c). Therefore, co-culturing of 786-O cells and HUVECs significantly enhanced the migration and invasion of 786-O cells in an MCTs-dependent manner.

### Co-culturing significantly increased the expression of MCT1 and MCT4 in both 786-O cells and HUVECs

**Fig. 4:**
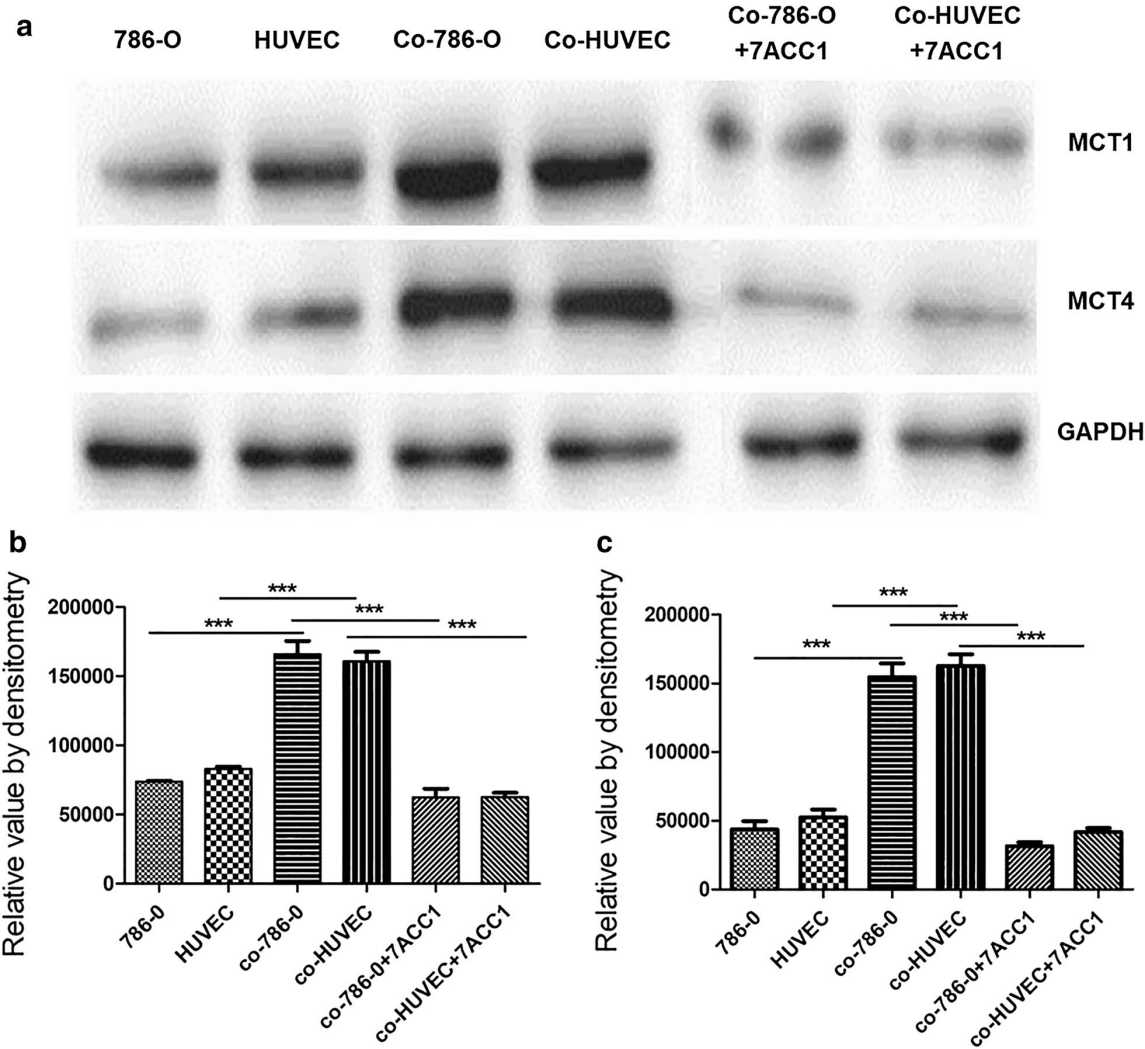
Co-culturing significantly increased the protein expression of MCT1 and MCT4 in both 786-O cells and HUVECs. **a**, **c** In transwell culturing, 1 × 10^4^ cells were seeded in the upper chamber and 4 × 10^4^ cells were seeded in the lower chamber. The protein levels of MCT1 and MCT4 in 786-O cells and HUVECs at 24 h after culturing were measured by western blotting. **a** Representative images show the protein levels of MCT1 and MCT4 in indicated cells at specified culturing conditions. **b**, **c** Summarized data shows the relative band intensity of (**b**) MCT1 protein (**b**) and (**c**) MCT4 protein in indicated cells in specific culturing conditions. For single culture, the cells were seeded in both the upper and lower chambers; in the HUVEC coculture, the 786-O cells were added to the upper chamber while HUVECs were added to the lower chamber; in the 786-O coculture, HUVECs were added to the upper chamber while 786-O cells were added to the lower chamber; in coculture + 7ACC1, 10 µM 7ACC1 was added to the culture medium during co-culturing. ***P < 0.001, between the indicated groups

**Fig. 5:**
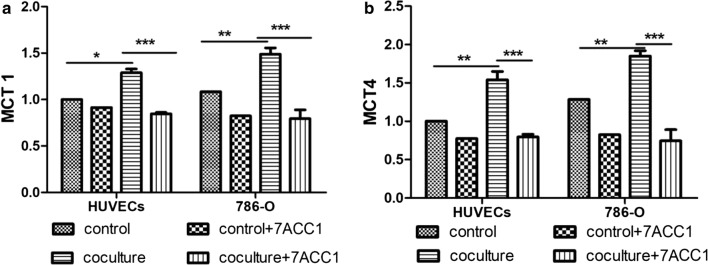
Co-culturing significantly increased the mRNA levels of MCT1 and MCT4 in both 786-O cells and HUVECs. **a**, **b** In the transwell culturing, 1 × 10^4^ cells were seeded in the upper chamber and 4 × 10^4^ cells were seeded in the lower chamber. The mRNA levels of (**a**) MCT1 and (**b**) MCT4 in 786-O cells and HUVECs at 24 h after culturing were measured by RT-qPCR. In the control, the cells were seeded in both the upper and lower chambers; in the HUVEC coculture, 786-O cells were added to the upper chamber while HUVECs were added to the lower chamber; in the 786-O coculture, HUVECs were added to the upper chamber while 786-O cells were added to the lower chamber; in control + 7ACC1 and coculture + 7ACC1, 10 µM 7ACC1 was added to the culturing conditions. *P < 0.05, **P < 0.01, ***P < 0.001, between the indicated groups

The expression levels of MCT1 and MCT4 in 786-O cells and HUVECs in different culturing modes were measured by western blot (Fig. 4) and RT-PCR (Fig. 5). Compared with cells in the control single-culturing condition, both 786-O cells and HUVECs showed significantly increased expression of MCT1 and MCT4 proteins (*P *< 0.0001; Fig. 4). However, at 24 h after incubation with the MCTs blocker 7ACC1, the protein expressions of MCT1 and MCT4 were attenuated to the similar levels as that in single culturing condition. Consistently, similar trends of mRNA expression of MCT1 (Fig. 5a) and MCT4 (Fig. 5b) in both 786-O cells and HUVECs were identified. Collectively, these results demonstrated that co-culturing of 786-O cells and HUVECs significantly increased the expression of MCT1 and MCT4, which could be abrogated by the MCTs inhibitor 7ACC1.

### Co-culturing of 786-O cells and HUVECs significantly increased extracellular lactate

**Fig. 6:**
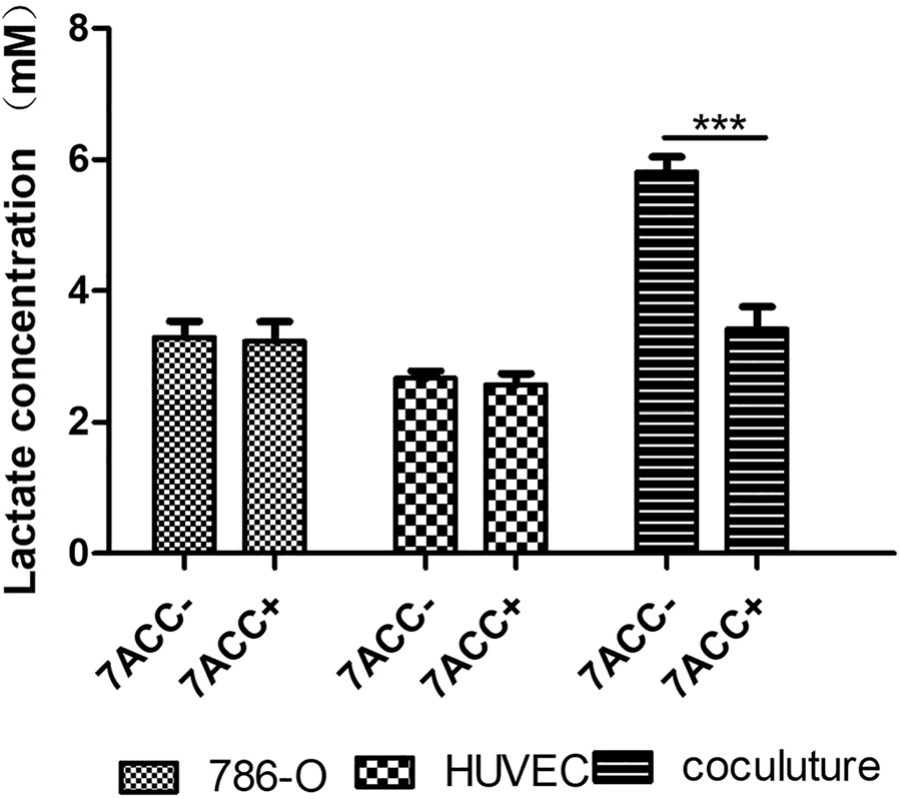
Co-culturing of 786-O cells and HUVECs significantly increased extracellular lactate. In the transwell culturing, 1 × 10^4^ cells were seeded in the upper chamber and 4 × 10^4^ cells were seeded in the lower chamber. The extracellular lactate was measured at 24 h after culturing in the absence or presence of 10 µM 7ACC1. In the single culture, cells were seeded in both the upper and lower chambers; in the coculture, 786-O cells were added to the upper chamber while HUVECs were added to the lower chamber. ***P < 0.001, between the indicated groups

To further confirm the role of MCTs in the co-culturing mode, we determined the ability of MCT1 and MCT4 to downregulate lactate uptake through measuring the extracellular concentration of lactate. As shown in Fig. 6, the lactate level was significantly higher in culture media from the co-culturing mode (5.81 ± 0.29 mM) than that from the single-culturing mode of 786-O cells (3.29 ± 0.25 mM) or HUVECs (2.7 ± 0.65 mM; *P *< 0.001). 24 h after MCT1 and MCT4 were blocked by 7ACC1, the extracellular lactate level (3.42 ± 0.34 mM) was significantly down-regulated, compare with that in the co-culturing mode (*P *< 0.001). However, lactate uptake did not show significant difference in 786-O cells or HUVECs from the single-culturing mode, when comparing cells treated with or without 7ACC1 (Fig. 6). Therefore, co-culturing of 786-O cells and HUVECs enhanced the activity of MCT1 and MCT4 to produce more lactate, which was also confirmed to be attenuated by the 7ACC1 treatment.

## Discussion

Most solid tumors depend on glycolysis for energy production, while glycolysis contributes to the acidic microenvironment of the tumor [21]. Studies in multiple types of cancers have unraveled the association between the upregulated expression of monocarboxylate transporters, MCT1 and MCT4, with the increased tumor malignancy and poor patient prognosis [3, 15, 16, 18, 20]. High expression of MCT1 or MCT4 is well correlated with worse prognosis in patients with ccRCC. Thus, MCT1 and MCT4 represent the biomarkers that can be used for predicting the prognosis of patients with ccRCC [3, 22–25]. In this study, we demonstrated that MCT1 and MCT4 expression was significantly increased in both 786-O cells and HUVECs during co-culturing, and their up-regulation contributed to the proliferation, migration, and invasion of 786-O cells.

In solid tumors, endothelial cells contribute significantly to tumor progression and account largely for the intrinsic aggression of the disease [26, 27]. Endothelial cells are surrounded by a high lactic acid environment, which can activate numerous signaling pathways and promote endothelial cell proliferation [28, 29]. Studies have demonstrated that endothelial cells rely on glycolysis for ATP production and cause a series of molecular events such as increased invasive activity of tumors [30, 31]. Therefore, we established an in vitro model in which renal cancer cells and endothelial cells were co-cultured. Our results demonstrated that the viability of renal cancer cells and endothelial cells was significantly higher in the co-culturing mode than in the single-culturing mode. Furthermore, the expression levels of both MCT1 and MCT4 were remarkably increased in either 786-O cells or HUVECs in the co-culturing mode, compared with the cells in the single-culturing mode. More importantly, 786-O cells showed enhanced migration and invasiveness in the co-culturing mode, which could be attenuated by the MCT blocker 7ACC1.

We found that 7ACC1 could significantly decrease MCT1 and MCT4 expression in 786-O cells and HUVECs when they were co-cultured. However, 7ACC1 did not exhibit a strong effect on downregulating MCT1 and MCT4 expression when 786-O cells and HUVECs were single cultured. Therefore, we hypothesize that 7ACC1 might inhibit the synthesis of MCT1 and MCT4 mRNA and/or protein translation with the help of cytokines secreted from endothelial cells. However, the related soluble factors and their roles in this process remain unclear so far. It has been reported that 7ACC1 can bind to the shMCT sites of MCT1 and MCT4 to block lactate efflux, thus attenuating the expression of MCT1 and MCT4 [20, 32]. It is worth further addressing the mechanisms underlying 7ACC1 induced suppression on MCT1 and MCT4 expression in our future studies.

A previous report from Payen et al. showed that pharmacologic MCT1 inhibition did not inhibit the migration and invasion of SiHa human cervix adenocarcinoma cells and 4T1 mouse mammary carcinoma cells in the single-culturing mode [33]. They proposed that MCT1 was not an essential contributor to pH-dependent tumor invasion. However, the tumor vascular microenvironments and the interactions between cancer cells and endothelial cells cannot be neglected in tumor metastasis. Recently, functional characterization in growing tumors revealed that MCTs are not only gatekeepers of intercellular metabolic cooperation but also important regulators of angiogenesis [34]. Lactate that enters into oxygenated endothelial cells and oxidative cancer cells via MCT1 promotes angiogenesis as it acts as a hypoxia-mimetic that activates the two transcription factors, hypoxia-inducible factor-1α (HIF-1α) and nuclear factor-κB (NF-κB), thereby stimulating the production of proangiogenic agents, like vascular endothelial growth factor (VEGF), basic fibroblast growth factor, and interleukin-8 (IL-8) [35]. Our study further underscored the critical roles of MCT1 and MCT4 in lactic acid transportation and tumor metastasis under cancer-endothelial cell co-culturing conditions.

The molecular mechanisms underlying the Warburg effect are pleiotropic, involving defects and gain-of function modulations of glycolytic and mitochondrial enzymes. These mechanisms might differ between types of cancer and even between cell lineages within a tumor [33]. Our study demonstrated that under co-culturing conditions, RCCs and endothelial cells could promote each other by upregulating the level of glycolysis for ATP production and increase the invasive activity of tumors. To alleviate pH stress in tumor cells due to acidification, MCT1 and MCT4 can be potently activated to regulate the uptake and release of lactate from tumor cells, which renders the endothelial cells adapted to acidification in the tumor microenvironment [36]. We found that 786-O cells or HUVECs in single-culturing conditions had lower MCT1 and MCT4 expression than the cells in co-culturing conditions. The lactate level was significantly higher in the culture media of the co-culturing mode than that in single-culturing of 786-O cells or HUVEC cells, which suggested that the lactate flux was up-regulated between cancer and endothelial cells.

Some signal transduction pathways contribute to the Warburg effect and the metabolic phenotype of cancer cells. For example, signaling initiated by growth factors results in the activation of PI3K/Akt and Ras via RTKs [37]. Akt increases glucose transporter activity and promotes glycolysis by activating several glycolytic enzymes, including hexokinase and phosphofructokinase (PFK). Phosphorylation of apoptotic proteins (such as Bax) by Akt makes cancer cells resistant to apoptosis and helps stabilize the mitochondrial outer membrane (OMM) by promoting the attachment of mitochondrial hexokinase (mtHK) to the VDAC channel complex [38]. Signal transduction of RTK to c-Myc induces the transcriptional activation of numerous genes that are involved in glycolysis and lactic acid production. The p53 oncogene transactivates the Tp53-induced Glycolysis and Apoptosis Regulator (TIGAR) and leads to an increase in NADPH production by PPS [39]. In addition, signaling from hypoxia-inducible factor (HIF) can increase the expression of LDHA to promote lactic acid production and the expression of pyruvate dehydrogenase kinase, thereby inhibiting the activity of pyruvate dehydrogenase and limiting the entry of pyruvate into tricarboxylic acid cycle [40]. Whether and how these signaling pathways interact with MCT1 and MCT4 in RCC remains to be elucidated in our further studies.

It has been shown that human tumors-specific lactate accumulation is correlated with metastasis, tumor recurrence, and poor survival of patients [41, 42]. Rather than a metabolic dead-end product of glycolysis, lactate is more like a tumor growth-promoting factor. Oxidative cancer cells utilize lactate in mitochondrial metabolism as a major fuel, while glycolytic cancer cells depend on glucose as a major fuel. Therefore, oxidative cancer cells with imported lactate can spare glucose for glycolytic cancer cells [21]. Moreover, strong evidence suggests that the glycolytic phenotype confers a significant growth advantage in cancer cells [21]. MCT4 shows a higher K_m_ for pyruvate than lactate, and this helps prevent the efflux of pyruvate and maintain a relatively low glycolytic flux. Conversely, MCT1 has a high affinity for lactate and helps the uptake of lactate in oxidative cancer cells [43]. In this study, the same trend observed for MCT1 and MCT4 expression is also observed in RCCs. Whether and how MCT1 and MCT4 contribute independently to the pathogenesis of renal carcinoma remain to be investigated with additional loss-of-function studies.

At present, several MCT inhibitors, including 7ACC2 [20], AZD3965a [44], and AR-C155858 [45, 46] have been developed. A single-MCT specific inhibitor, such as MCT1-specific AZD3965, might also be useful for directly delineating the independent role of MCTs in RCCs. In our study, 7ACC1, a blocker of MCTs was used to inhibit lactate transportation. The complementation of 7ACC1 in the culture medium significantly slowed cell proliferation, migration, and invasion of 786-O cells in co-culturing conditions. Similarly, lactate uptake showed no significant difference during the single-culturing of either 786-O cells or HUVECs treated with and without 7ACC1 but was significantly inhibited in co-cultured cells that were treated with 7ACC1. These results are interesting and unexpected. When tumor cells coexist with vascular endothelial cells in hypoxic and acidic environments, the energy metabolic pathway of glycolysis is activated, which is critical for tumor growth and angiogenesis [12]. Glycolysis is enhanced by MCT1 and MCT4, and therefore, reprogramming of glucose metabolism occurs [11]. In this metabolic model, lactate is produced and utilized as a main energy resource by both cancer and endothelial cells. Therefore, it is plausible that targeted blocking of lactate transporters can impair the viability and migration ability of both 786-O cells and HUVECs, as well as the invasion ability of 786-O cells.

## Conclusions

Our results suggest that MCT1 and MCT4 play a central role in renal cancer metabolism. Compared with the single-culturing mode, 786-O cells co-cultured with HUVECs displayed significantly enhanced proliferation, migration, and invasion. Our work demonstrated that the Warburg effect accompanied with high MCT1/MCT4 expression in cancer-endothelial microenvironments contributed significantly to renal cancer progression. Targeted blocking of MCT1 and MCT4 can downregulate lactate flux, thereby inhibiting the proliferation and invasive ability of renal cancers.

## Data Availability

The datasets used and/or analyzed during the current study are available from the corresponding author on reasonable request.

## Abbreviations

RCC: renal cell carcinoma
ECs: endothelial cells
MCTs: monocarboxylate transporters
ccRCC: clear cell renal cell carcinoma
BCA: bicinchoninic acid
SDS-PAGE: sodium dodecyl sulfate polyacrylamide gel electrophoresis
PVDF: polyvinylidene difluoride
BSA: bovine serum albumin
RT-qPCR: reverse transcription-quantitative polymerase chain reaction
HIF-1α: hypoxia-inducible factor-1α
VEGF: vascular endothelial growth factor
IL-8: interleukin-8
